# Diagnostic, Therapy and Complications in Acute Appendicitis of 19,749 Cases Based on Routine Data: A Retrospective Multicenter Observational Study

**DOI:** 10.3390/jcm11154495

**Published:** 2022-08-02

**Authors:** Claus W. Schildberg, Kathrin Reissig, Richard Hunger, Christoph Paasch, Rosi Stillger, René Mantke

**Affiliations:** 1 Department of Surgery, Brandenburg Medical School, University Hospital Brandenburg/Havel, 14770 Brandenburg, Germany; reissig.mhb@klinikum-brandenburg.de (K.R.); richard.hunger@mhb-fontane.de (R.H.); paasch.mhb@klinikum-brandenburg.de (C.P.); chirurgie@klinikum-brandenburg.de (R.M.); 2 CLINOTEL Hospital Association Gemeinnuetzige GmbH von- der- Wettern-Str.27, 51149 Köln, Germany; stillger@clinotel.de; 3 Faculty of Health Science Brandenburg, Brandenburg Medical School, University Hospital Brandenburg/Havel, 14770 Brandenburg, Germany

**Keywords:** 19,749 appendectomies, CT gained importance, avoid unnecessary operations

## Abstract

Background: Acute appendicitis is one of the most common emergencies in general surgery. The gold standard treatment is surgery. Complications may occur during or after an appendectomy. In addition to age, clinically important factors for the outcome after appendicitis seems to be the comorbidities and the stage of the appendicitis at the time of the operation. Large observational data describing these facts are missing. Methods: In this retrospective multicenter observational study, all inpatients over the age of 17 years with a diagnosis of acute appendicitis in 47 hospitals of the Clinotel Hospital Group between 2010 and 2017 were included. Results: A total of 19,749 patients with acute appendicitis were operated on. The number of patients with more than five secondary diagnoses has increased from 8.4% (2010) to 14.5% (2017). The number of secondary diagnoses correlates with the ages of the patients and leads to a significantly longer hospital stay. Computer tomography (CT) has gained in importance in recent years in the diagnosis of acute appendicitis. A total of 19.9% of patients received a CT in 2017. Laparoscopic appendectomy increased from 88% in 2010 to 95% in 2017 (*p* < 0.001). The conversion rate did not change relevant in the study period (i.e., 2.3% in 2017). Appendicitis with perforation, abscess, or generalized peritonitis was observed in 24.8% of patients. Mortality was 0.6% during the observation period and was associated with age and the number of secondary diagnoses. The analysis is based on administrative data collected primarily for billing purposes, subject to the usual limitations of such data. This includes partially incomplete clinical data. Conclusions: Multimorbidity is increasingly present in patients with acute appendicitis. Mortality is still in an acceptably low range with no increase. A CT scan is necessary for a precise diagnosis in unclear clinical situations to avoid unnecessary operations and was performed more often at the end of the study than at the beginning.

## 1. Introduction

Acute appendicitis is a common surgical abdominal emergency and the main cause of an acute abdomen in more than 20% of cases [1,2,3,4]. Appendectomy is the treatment of choice in most cases and therefore the most common visceral surgical emergency intervention. About 135,000 patients undergo appendectomy in Germany every year [5]. Lifetime incidence is 8% with a peak of disease between 20 and 40 years of age [2]. In recent years in Germany, some studies have addressed the outcome or risk factors of morbidity after appendectomy [3,5,6]. Furthermore, the surgical management of acute, complicated, or uncomplicated appendicitis, as well as the timing of surgery, has been analyzed [7]. Nevertheless, there is a lack of current studies with a great number of enrolled individuals. This study aims to analyze the development of epidemiology, diagnostics, the type of surgical techniques, and mortality in acute appendicitis in 47 hospitals in Germany based on routine data between 2010 and 2017. This study is a retrospective multicenter observational study.

## 2. Methods

### 2.1. CLINOTEL Hospital Association Germany 

The study was performed by means of administrative routine data of health insurance claims data, which were collected according to §301 SGB V and § 21 “Krankenhausentgeltgesetz (KHEntG)”. Data were provided by the CLINOTEL Hospital Group Germany and were derived from anonymous billing data for inpatient hospital treatment. Diagnoses were coded according to the International Classification of Disease, German modification coding guideline [8]. Procedures were documented based on the German version of the International Classification of Procedures in Medicine (OPS) [9]. The German Diagnosis-Related Groups System (DRG) is jointly addressed to German healthcare providers and healthcare insurance schemes for billing claims. The CLINOTEL Hospital Association Germany covers 66 small local, large local, county, or regional/university teaching hospitals with 60 departments of general surgery in Germany. With respect to the main diagnosis, 47 hospitals could be included, from which more than 4 million patients (n_all_ = 4,020,039) had been discharged in 2010–2017. The work has been reported in line with the STROCCS criteria [10].

### 2.2. Patients and Data

**Figure 1:**
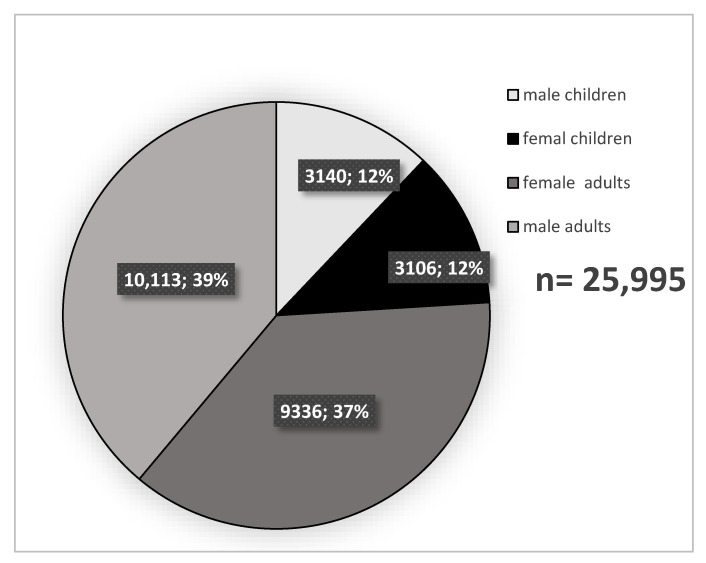
Number of all patients with acute appendicitis, 2010–2017 (*n* = 25,995).

All inpatients with a diagnosis of acute appendicitis (ICD-10-GM, K35) were included in the study. Patients treated conservatively with antibiotic therapy were not included in the study. The average number of annual cases of acute appendicitis varied from 4 to 271 patients per hospital. Between 2010 and 2017, the 47 hospitals in the study cohort surgically treated 25,995 patients diagnosed with acute appendicitis (Figure 1).

All surgically treated patients including pregnant women or immunocompromised patients above 17 years of age from 2010–2017 with a main discharge diagnosis of acute appendicitis K35 according to ICD-10-GM (*n* = 19,749) were identified. Appendicitis was defined based on histological findings. Cases with other types of appendicitis (ICD-10-GM, K36) or patients who underwent appendectomy in the context of other operations were not included. Up to 6 diagnoses and 3/19 procedure codes, as well as up to 5 secondary diagnoses and 14 different common complications (ICD-10-GM, T80 to T88) were recorded. The length of stay was determined from the admission and discharge dates, as the date of the operation was not recorded. Common complications are jointly defined according to the quality assurance with routine data, ICD-10-GM and OPS guidelines. In this study, comparisons were made of sex, age, number of secondary diagnoses, and of CT scans, surgical methods, and common complications. In a second step, comorbidity was analyzed. For this purpose, the number of secondary diagnoses was broken down more precisely. 

### 2.3. Statistical Analysis

The descriptive analysis was performed using mean and standard deviation for continuous and frequencies or percentages for categorical variables. Associations between categorical variables were assessed by Chi-square tests and temporal trends by Cochran–Armitage tests. Statistical analyses were performed using two-sided tests with the aid of SPSS software 26.0 (Amaronk, NY, USA: IBM Corp.), and *p*-values of <0.05 were considered to indicate significant statistical effects.

The study has been registered under the following number: researchregistry7811 (https://www.researchregistry.com/browse-the-registry#home/registrationdetails/6258118f44a719001ea620b2/ accessed on 14 April 2022).

## 3. Results

### 3.1. Study Population

Between 2010 and 2017, a total of 19,749 adult patients with a main diagnosis of acute appendicitis were subjected to surgical treatment in 47 hospitals of the CLINOTEL Hospital Association in Germany. A total of 6246 underage patients (24%) were excluded from the analysis. 

**Figure 2:**
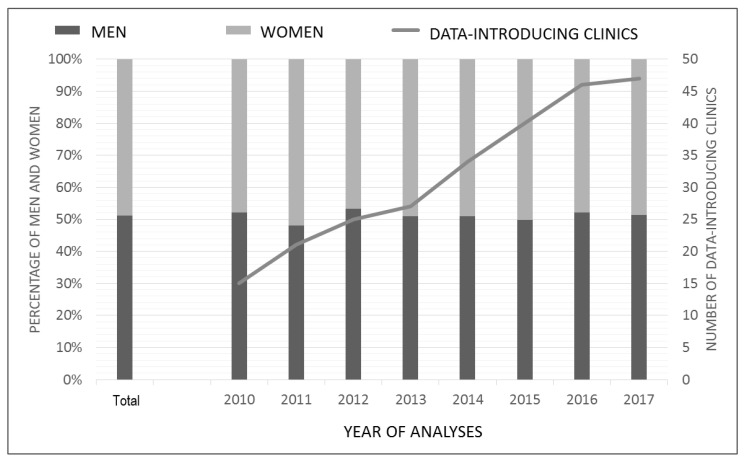
Gender distribution of all appendicitis cases (adults) and number of data introducing clinics, 2010–2017 (*n* = 19,749).

The gender distribution of patients with a main diagnosis of acute appendicitis in each study year is shown in Figure 2 and remained unchanged during the study period (*p* = 0.58). The overall male:female ratio was 1.05, indicating a slightly higher incidence in males (51.2% 95%-CI: [50.5, 51.9], *p* < 0.001). Additionally, the figure displays the increasing number of participating hospitals during the study period. On average, 79.7 (SD = 40.5, range: 1–196) patients were treated per hospital annually. The mean age was 39.8 years for male and 39 years for female patients. The most patients with acute appendicitis were observed in the ages from 18 to 29 years (41.9%, *n* = 8270, Table 1).

### 3.2. Diagnostic and Clinical Presentation

**Figure 3:**
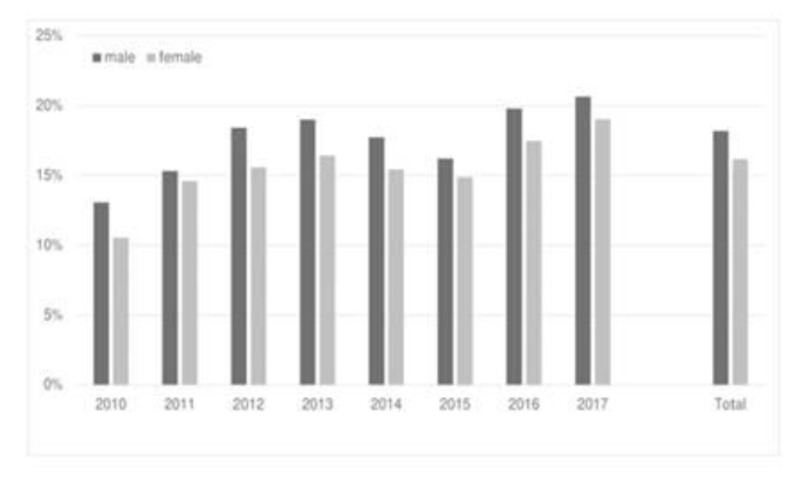
Performed CT scans (male/female, %) to detect the diagnosis of appendicitis, 2010–2017 (*n* = 19,749).

First, we examined the number of performed diagnostic CT scans. On average, a CT was performed in 17.2% of the patients. In 2010, a CT scan was conducted in 11.9% of cases, while in 2017 the percentage increased to 19.9% (*p* < 0.001; Figure 3). The number of patients who underwent MRI scans was 0.6%. The frequency of ultrasound examinations cannot be shown based on the routine data, as these are not documented.

**Figure 4:**
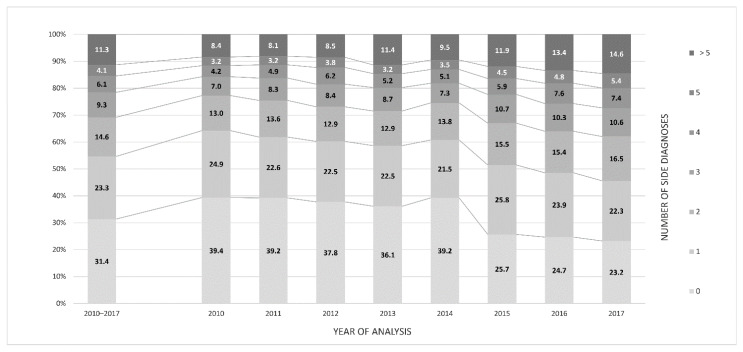
Number of secondary diagnoses accompanying a main diagnosis of acute appendicitis in all appendectomy cases, 2010–2017 (*n* = 19,749).

The secondary diagnoses were mainly cardiopulmonary in nature and increased with age. The number of secondary diagnoses by study year is depicted in Figure 4. The proportion of cases with only one secondary diagnosis decreased significantly from 39.4% in 2010 to 23.3% in 2017 (*p* < 0.001). Correspondingly, the proportion with more than five secondary diagnoses increased significantly (*p* = 0.001) from 8.4% to 14.5% (Figure 4). A statistically relevant analysis of mortality over the years was not possible because only 46 patients died during the observation period.

**Figure 5:**
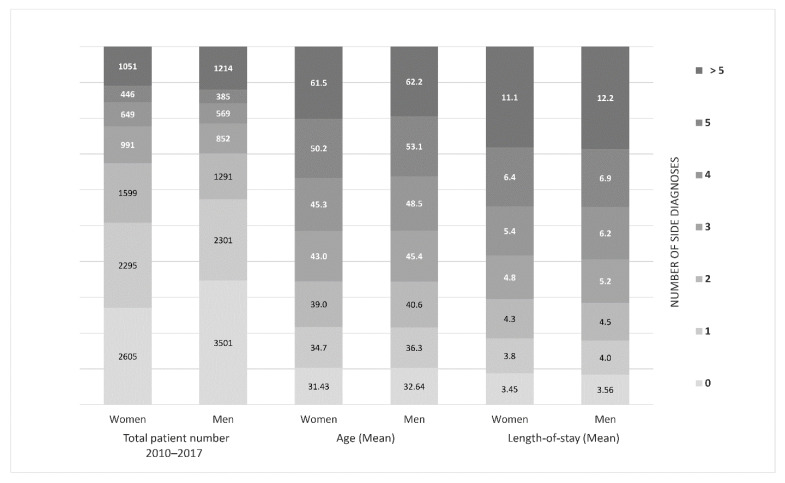
Number of secondary diagnoses: men compared to women. Mean age and mean duration of hospitalization of all patients with a main diagnosis of acute appendicitis in all appendectomy cases, 2010–2017 (*n* = 19,749).

A strong association between higher age and increasing number of side diagnoses was observed (*p* < 0.001, Figure 5). Adjusted pairwise comparisons between gender and the number of side diagnoses showed a significantly larger proportion of males had zero (male 32.6% versus female 31.4%), as well as more than five side diagnoses (male 62.2% versus female 61.5%) (*p* < 0.001 and *p* = 0.033, respectively). The mean age of the patients with more than five secondary diagnoses was 61.5 years for women and 62.2 years for men. With an increasing number of secondary diagnoses, patients had significantly longer hospitalization times (*p* < 0.001). The length of the inpatient stay in patients with more than five secondary diagnoses was 11.1 days in women and 12.2 days in men, a more than threefold increase compared to patients without secondary diagnoses (women 3.5 and men 3.6 days, Figure 5).

### 3.3. Therapy

**Figure 6:**
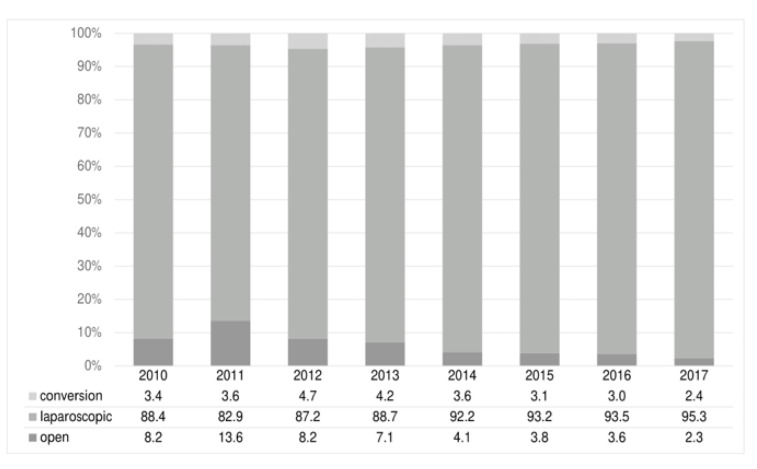
Operation type and conversation rate (%), 2010–2017 (*n* = 19,749).

The majority of operations were performed laparoscopically. The proportion of laparoscopic appendectomies increased significantly from 88% in 2010 to 95% in 2017 (*p* < 0.001). The conversion rate from laparoscopic to open appendectomy remained nearly the same (2.5–4.7, Figure 6). The main reason for switching to open surgery was the unclear situation during laparoscopy. 

**Figure 7:**
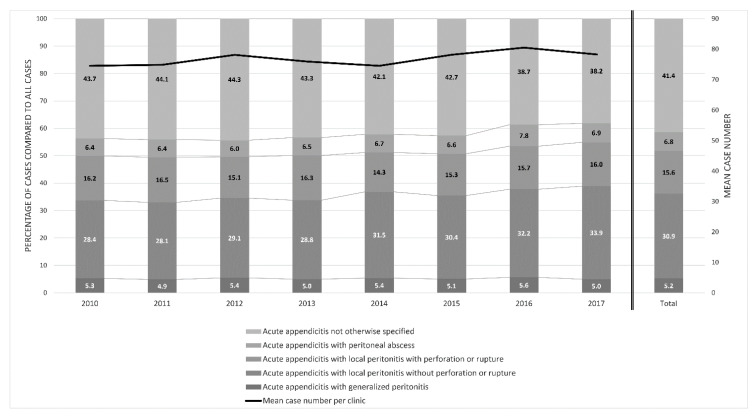
Distribution of cases with a main diagnosis of acute appendicitis by type of appendicitis (according to the ICD-10 guideline) between 2010 and 2017 (*n* = 19,749).

Regarding the type of appendicitis, the most common were acute not otherwise specified appendicitis (37.2%) and acute appendicitis with local peritonitis without perforation or rupture (27.8%), as shown in Figure 7. About 25% of all cases were attributable to more complicated forms of acute appendicitis: with local peritonitis and perforation or rupture (15.6%), with local peritonitis and peritoneal abscess (5.2%), or with generalized peritonitis (4.7%). There were no differences across the years (*p* = 0.09).

### 3.4. Complications

**Figure 8:**
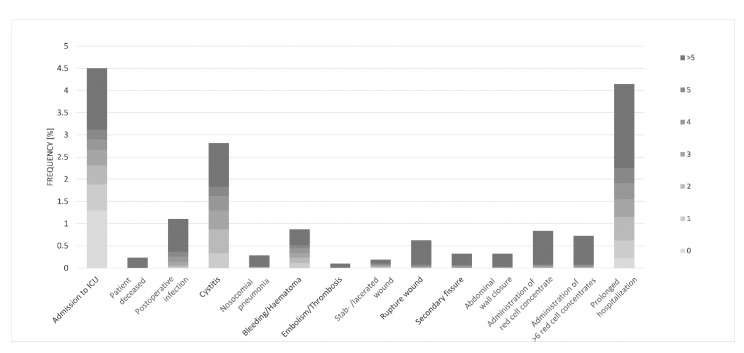
Type and frequency of complications. Recorded complications regarding the number of side diagnoses with main diagnosis acute appendicitis of all appendectomy cases (adults) in CLINOTEL Association between 2010 and 2017, *n* = 3374.

The overall complication rate was 17.1% (*n* = 3374, Figure 8) and was more frequently observed in females than in males (17.9% vs. 16.3%, *p* = 0,002). The most common general complications were admission to the intensive care unit (4.5%) and prolonged hospitalization (4.1%), followed by cystitis (2.8%), while the most common surgical complication was postoperative infection (1.1%). Ten further complications were rare, with incidence rates less than 1.0%. The total lethality was 0.6%. The lethality in male and female patients, as well as between laparoscopic and open surgery, was almost the same. There was a clear relationship toward elevated mortality with increasing age (*p* < 0.001). Likewise, a strong association was found between a higher number of comorbidities and increased mortality (*p* < 0.001).

## 4. Discussion

At first glance, acute appendicitis appears to be an everyday surgical diagnosis that is not the focus of scientific research. Appendicitis is currently moving into focus again because of the discussion of when uncomplicated acute appendicitis can be treated conservatively with antibiotics and painkillers without worsening the results [11,12]. To be able to conduct such discussions, the surgical results of the treatment of acute appendicitis must be clearly analyzed and also published in an up-to-date manner.

After the publication by Sahm et al., who analyzed the reality of care in Germany for acute appendicitis in the years 1988/1989, 1996/1997, and 2008/2009 with register data, we have now carried out analyses from 2010–2017 using routine data. Large population-based or register-based studies focusing on appendicitis are generally rare [13,14,15]. However, these studies are particularly important at describing the developments in diagnostics, therapy, and treatment results over the years.

### 4.1. Population and Diagnostic

We analyzed 19,749 adult patients based on German routine data from 47 hospitals. 

When comparing the gender distribution, there was no difference to other studies that showed men and women to be almost equally distributed [16,17]. The age distribution in our study with a significant increase in patients from 18–29 years (41.9%) and 30–39 years (16.7%) corresponds to the publications of other authors [18,19].

Acute appendicitis has a highly variable clinical presentation [11]. In addition to clinical examination, abdominal sonography is an important and universally available diagnostic method in Germany [20]. Sonography is therefore reliable to confirm the presence of appendicitis but unreliable to exclude appendicitis and it is highly operator-dependent [20]. However, the routine data we analyzed are not suitable for evaluating the frequency of use of sonography, since sonography is not documented for billing purposes. In contrast, we were able to make statements on the use of CT and MRI in the diagnosis of acute appendicitis. It is generally accepted that low-dose CT without oral contrast has a sensitivity of 76–100% and a specificity of 83–100% in the diagnosis of appendicitis and is therefore superior to sonography [20]. However, especially the use of standard-dose CT has led to a significant increase in ionizing radiation with the risk of cancer [21]. For this reason, in many clinics in Germany, computed tomography is only performed after a negative sonography. If acute appendicitis is detected in the sonography, the CT can be dispensed with. This is particularly important since a large proportion of the patients in our study are also relatively young. The scientific publications on the use of CT in the diagnosis of acute appendicitis have also led to an increase in CT examinations in Germany from 11.9% in 2010 to 19.9% in 2017. This coincides with the results of other studies in the literature [22,23,24,25,26,27,28,29,30,31,32,33,34,35,36]. The average proportion of cases that underwent CT was 20% in a non-American study, like our group, and even 90% in a large US study [37]. However, the above-mentioned 20% is considered too low in the current discussion as Mowbraw et al. [38] in a recently published British study showed that the number of unnecessary appendectomies fell from 22% to 7% with increasing CT (from 36.3% to 85.9%, *p* < 0.001) examinations. These results were additionally confirmed in a systemic review and in the WSES Jerusalem guidelines, so that more CT should be performed to avoid unnecessary operations [39,40]. It is therefore to be expected that there will be an increase, especially in low-dose CT examinations in the future, although there is no association between increased use of CT and more diagnosed perforated appendicitis [39]. Since many patients (41.9% in our study) are between the ages of 18 to 29, radiation exposure and the risk of cancer are always a topic of discussion. The use of a low-dose CT significantly minimizes this risk but still gives good radiological results [21,41]. MRI was only used to a very limited extent in our study (0.6%). However, the sensitivity and specificity of MRI in the diagnosis of acute appendicitis are comparably good with the CT, especially in the subgroups of pregnant patients and children [42]. When interpreting our results, however, it should be noted that we excluded children and pregnant women from the analysis of the MRI figures. From our point of view, it can be assumed that the proportion of MRI diagnostics will increase in the coming years. In addition to the higher costs compared to CT, the limitations here are the lack of 24-h/7-day availability of this method in many clinics in Germany. It is noteworthy that in our analysis there was a significant increase in secondary diagnoses over the study period, although 41.9% of our patients were aged between 18 and 29 years. Only 15% of our patients were older than 60 years. This trend toward surgery for patients with increased comorbidity has also been confirmed in other populations [16,35].

It is known from other analyses that the frequency and type of secondary diagnosis can also affect mortality [43,44]. However, we could not observe this in our study collective with appendicitis. On the other hand, the number of secondary diagnoses correlates with the age of the patients and leads to a significantly longer hospital stay in our analyses.

### 4.2. Therapy and Complications

Our data show a further increase in laparoscopic appendectomies over the observation period to 95% in 2017. This change from the open to the laparoscopic surgery is also described in the literature [45,46,47,48,49,50,51,52,53,54,55,56,57,58]. On the other hand, the conversion rate for open surgery was statistically constant and ranged only from 2.5 to 4.7%. It is interesting that there were no significant differences in the complication rates between open and laparoscopic surgery. Similar results are available in the literature [55,58]. Nevertheless, it is still recommended that the type of surgery chosen should be dependent on the relative experience of the surgeon with respect to the two methods [58]. However, one can assume that if 95% of the interventions are carried out laparoscopically, there is sufficient expertise in the method in all clinics. No statement can be made about the number of patients who were treated conservatively with antibiotic therapy because we did not include them in the study.

Perforated appendicitis did not change over time in our population and ranged from 14.3% to 16.2% (acute appendicitis with local peritonitis and perforation or rupture). Other authors described a trend toward more severe courses [16,31]. Some groups reported that perforations were more common in patients over 50 years of age [6,56,59]. Our overall complication rate was reasonable at 17% and comparable to the outcome of other study groups. Other groups also reported a similar rate of postoperative wound infections (1%) [46,58]. The increase in serious complications that were due to advanced inflammation and the correlation between a higher number of comorbidities and increased mortality has been published by other authors [47,59]. Mortality in our observational study also corresponds to other published rates [46,56,59]. Masoomi et al. (2012), for example, reported a mortality from laparoscopic appendectomies of 0.7% [60]. Likewise, a strong association was found between a higher number of comorbidities and increased mortality (*p* < 0.001).

Because the analysis is based on administrative data collected primarily for billing purposes, these have the usual limitations of such data. This includes, above all, partially incomplete clinical data. In addition, it should be noted that the hospital network mainly comprises medium-sized hospitals. However, since surgical treatment of appendicitis is part of the standard repertoire of general surgery departments, no systematic bias with respect to a particular case severity is to be expected. Certainly, a more specific analysis of the clinical characteristics of the patient population in whom CT was performed would be interesting, especially in comparison to the non-CT group. However, this analysis was not possible because of the administrative nature of the data.

## 5. Conclusions

Appendicitis has been and remains a very common surgical emergency that is almost equally common in women and men. The CT scan is becoming increasingly important, especially for a more precise diagnosis in the case of unclear clinical manifestations and to avoid unnecessary operations, even in Germany. An average of 17% CT scans seems too few, since an increase can reduce the number of unnecessary appendectomies. Laparoscopic appendectomy is the standard procedure with constant low conversion rates. Due to the increase in comorbidity, more complex courses with extended hospital stays can be expected in the future.

## Figures and Tables

**Table 1:** Age distribution of adult patients with acute appendicitis, 2010–2017 (*n* = 19,749).

Age	*n*	%
18–29	8270	41.9
30–39	3292	16.7
40–49	2655	13.4
50–59	2564	13
60–69	1474	7.5
70–79	1053	5.3
80–89	400	2
90–99	41	0.2
	19,749	100
